# Caring Under Pressure: Investigating Parental Attitudes in Mother–Child Chronic Illness Dynamics

**DOI:** 10.3390/children11111348

**Published:** 2024-11-03

**Authors:** Melda Celik, Esma Altinel Acoglu, Beril Aydin, Emel Isiyel, Siddika Songul Yalcin

**Affiliations:** 1 Department of Pediatrics, Division of Social Pediatrics, Faculty of Medicine, Hacettepe University, 06230 Ankara, Türkiye; melda.celik@hacettepe.edu.tr; 2 Department of Pediatrics, Sami Ulus Maternity and Children’s Health and Diseases Training and Research Hospital, 06010 Ankara, Türkiye; 3 Department of Pediatrics, School of Medicine, Baskent University, 06490 Ankara, Türkiye; 4 Department of Pediatrics, Faculty of Medicine, Hacettepe University, 06230 Ankara, Türkiye

**Keywords:** child, chronic illness, mother, parental illness, parental attitudes

## Abstract

Background: The presence of chronic illnesses in both a mother and a child poses a significant challenge for mothers in managing these conditions, yet how maternal attitudes adapt to dual illness remains poorly understood. This study aims to explore parenting styles in families dealing with mother–child chronic illnesses. Methods: Mothers of children aged 2–6 were recruited from three pediatric clinics and categorized based on the health status of both the mother and the child. Data collection included case files and the Parental Attitude Scale (PAS), which assessed democratic, authoritarian, overprotective, and permissive attitudes. The interaction between mother–child health status and higher levels of parental subscales was analyzed using the chi-square test. Multiple logistic regression analysis was then performed to evaluate this interaction, controlling for confounding baseline characteristics. Results: In total, 878 mother–child pairs were included. Mothers exhibited varying attitudes based on education, employment, and the child’s age. Chronic illness in both mother and child and only the child having an illness were significantly associated with higher overprotective scores (*p* < 0.001). The percentage of mothers with high permissive scores was higher when only the mother had an illness and when both were healthy, compared to the case of both mother and child having an illness (*p* = 0.018). After adjusting for confounding factors, having a sick child showed a 1.6-fold increase in the likelihood of a high overprotective score, and both the mother and child having an illness showed a 2.94-fold increase. Similarly, after adjusting for the same confounding factors, the likelihood of a high permissive score was 2.56 times lower when both were ill compared to when both were healthy. Conclusions: This study reveals that when a child is affected by a chronic illness—whether or not the mother is also ill—mothers tend to exhibit higher levels of overprotection and lower levels of permissiveness, while their levels of authoritarianism and democratic attitudes in parenting remain relatively stable.

## 1. Introduction

Chronic illnesses or medical conditions are long-term health issues that persist for at least three months, disrupting the everyday life of the patient and their family and often requiring ongoing medical attention such as frequent hospital visits, home care, or specialized treatment [1]. Managing chronic illnesses in children presents a widespread challenge across the globe, encompassing a variety of physical conditions like asthma, diabetes, congenital heart disease, and chronic renal disease, as well as mental health conditions such as attention deficit hyperactivity disorder, with nearly 30% of children worldwide facing such conditions [2,3]. Chronic illnesses in children affect not only their physical and emotional well-being but also their daily functioning, with significant impacts on the entire family. Families provide critical support, especially during periods when the child is managing a chronic condition. Such illnesses can disrupt family life, altering dynamics and creating challenges such as the loss of routine, uncertainty, and strained relationships. The duration and severity of the illness, along with the type of care needed and the parents’ emotional and financial resources, greatly influence parental well-being [3,4,5,6,7,8,9]. In turn, parental stress and family dysfunction can negatively impact a child’s developmental outcomes, underscoring the importance of understanding these interconnected factors. Over time, these factors may contribute to shifts in parenting attitudes and behaviors as parents adapt to the ongoing demands of managing their child’s health condition. Therefore, adaptive parenting strategies, particularly those focusing on the parent–child relationship, are crucial in helping children cope with chronic illness [3,10,11,12].

At the same time, a significant percentage of parents suffer from chronic medical conditions, with global estimates ranging from 4% to 12% [13,14]. Ill parents’ children predominantly feel restricted in daily activities, isolating themselves from peer groups [15]. These children frequently struggle with the demands of caregiving and may lack critical information about their parent’s condition [13,16]. Research by Pierce et al. highlighted that children of ill parents had a lower quality of life, lower life satisfaction, and more psychosomatic complaints than their peers without parental illness [17]. A review of 41 studies revealed that persistent mental health disorders in parents, especially mothers, were associated with increased occurrences of child malnutrition and diarrhea in low- and middle-income countries, and injuries and asthma in others [18]. A meta-analysis of 19 studies on the differences in problem behaviors between children with chronically ill parents and those with healthy parents found significant overall effect sizes for internalizing and externalizing problem behaviors in children [14]. During the early developmental stage of children, mothers are typically the primary caregivers, making their health and well-being crucial in the child’s emotional and physical development [19,20]. Chronic illness in mothers can affect the dynamics of daily caregiving and the mother’s ability to fulfill caregiving responsibilities [21,22,23]. Understanding how maternal health impacts parenting strategies in this context is essential in developing targeted interventions that support both mothers and their children. Despite this, there is a significant gap in research exploring how parental illness shapes chronically ill parents’ attitudes toward their children. In addition, to our knowledge, there is a lack of literature addressing parental attitudes in the context of both a mother and a child experiencing chronic illness. This gap highlights the need for further investigation into how dual chronic illness impacts parenting behaviors and attitudes, offering opportunities for targeted interventions to support families facing such challenges.

Parenting styles and the quality of the parent–child relationship are crucial to children’s psychological development, particularly when dealing with chronic illness, as these factors can influence adherence to treatment [3,5]. Baumrind’s framework outlines main parenting styles—authoritarian, democratic, and permissive—each reflecting different levels of control, responsiveness, and autonomy. Democratic parenting fosters respect and independence, while authoritarian parenting emphasizes obedience and conformity, and permissive parenting allows freedom with minimal guidance. Authoritarian parents often prioritize maintaining authority, sometimes quickly suppressing challenges from their children. Permissive parents, on the other hand, tend to grant more autonomy, exercise little control, and, at times, show a degree of tolerance that may verge on neglect [24]. Overprotective parenting, marked by excessive support and developmentally inappropriate behaviors, limits children’s ability to handle age-appropriate challenges [25]. The attitude and behavior of parents might be different in situations where a child’s or their own chronic disease emerges.

Family Systems Theory and Stress-Vulnerability Theory provide a valuable framework for understanding how parenting styles evolve in the context of mother–child chronic illnesses [26,27,28]. According to Family Systems Theory, chronic illness can disrupt family dynamics, leading to changes in parental roles and behaviors. Stress-Vulnerability Theory further suggests that chronic illness introduces significant stress, which can impact a parent’s emotional resilience and result in shifts in their parenting approach. Together, these theories offer insights into how both family dynamics and individual stress responses influence parenting styles in the face of chronic illness.

This study hypothesized that both mothers of children with chronic illnesses and mothers having chronic illness would display higher levels of overprotectiveness and lower levels of permissiveness compared to typically healthy mothers whose children are not affected by chronic illnesses, while their authoritarian and democratic parenting attitudes would remain stable regardless of their child’s health status. This study seeks to examine different parenting styles—authoritarian, democratic, permissive, and overprotective—in families where both the mother and child are coping with chronic illnesses. It will explore the challenges that these families face and how maternal attitudes may either mitigate or exacerbate those challenges. The findings will provide valuable insights to help develop interventions that foster resilience and support positive outcomes for families navigating the complex realities of chronic illness.

## 2. Materials and Methods

### 2.1. Participants

In this study, we recruited mothers of children aged between 2 and 6 years from various pediatric clinics in Ankara, Turkey, from March to August 2022. Participants were sourced from three distinct healthcare facilities: Hacettepe University Children’s Hospital, Dr. Sami Ulus Children’s Health and Diseases Training and Research Hospital, and Baskent University Hospital. These hospitals were chosen to represent diverse healthcare settings, including a university hospital, a training and research hospital, and a private hospital, thus ensuring a broad representation of the population seeking medical care for their children.

This study included only children living with their mothers and mothers with at least one child aged 2 to 6 years. Participants were then grouped into four categories based on the health status of both the mother and the child: (a) Mother Ill, Child Healthy, where mothers had a chronic illness but their children were healthy; (b) Mother Healthy, Child Ill, where mothers were free from chronic illness but their child had at least one chronic illness; (c) Both Mother and Child Ill; and (d) Both Mother and Child Healthy. Upon enrollment, participating mothers provided informed consent to participate in the study.

### 2.2. Study Design

In this study, we utilized a 27-item case file created by the researchers to collect data on mother–child pair characteristics [29,30,31]. Additionally, we incorporated the “Parental Attitude Scale (PAS)” developed and validated by Demir and Şendil in 2008 [32]. This comprehensive tool consists of 46 items aimed at evaluating parental attitudes across four key dimensions: democratic, authoritarian, overprotective, and permissive. Each dimension offers insight into different aspects of parental behavior, such as responsiveness, control, warmth, and autonomy. The internal consistency of the PAS subscales, assessed by Cronbach’s alpha, was 0.83 for the democratic subscale, 0.76 for the authoritarian subscale, 0.75 for the overprotective subscale, and 0.74 for the permissive subscale in the previous study [23]. In our study, the Cronbach’s alpha values were 0.80, 0.69, 0.76, and 0.70, respectively.

### 2.3. Sample Size

With an effect size of 0.25 (medium), alpha error of 0.05, power of 0.95, 4 groups, and 9 covariate variables, the total sample size was calculated as 840. Adjusting for a 10% dropout rate and potentially faulty surveys, the target was set at 924 completed surveys.

### 2.4. Statistical Analysis

Data were analyzed using IBM-SPSS 23. The normality of variables was assessed using the Kolmogorov–Smirnov test. The mean value with standard deviation was given in the groups with normal distribution, and the median with quartiles was given for non-normal distributed parameters. PAS subscales showed skewed data. The highest quartile of PAS subscales from a previous study involving healthy mother–child pairs was used to define the upper (high) level for these subscales in the present study [29].

The PAS subscales were considered dependent variables, while mother–child pair characteristics were treated as independent variables.

Group differences in parameters with categorical data were assessed using the Chi-square test. When significant interactions emerged, subgroup analyses with residual analysis were conducted to identify the specific groups responsible for the differences.

The characteristics of mother–child pairs and the occurrence of high PAS subscale levels were evaluated using multiple logistic regression, adjusting for potential confounders. Variables with a *p*-value < 0.2 in the univariate analysis were considered confounding factors in the multivariate analysis. The relationship between mother–child variables and the high level on the PAS subscale score was examined in Model 1, while in Model 2, other PAS subscale scores were also included. Odds ratios (ORs) and 95% confidence intervals (CIs) were calculated, with statistical significance set at *p* < 0.05.

## 3. Results

During the study, out of the 930 surveys completed, 32 were missing data, and 20 did not meet the age criteria, resulting in 878 surveys being included in the analysis.

The participating mothers’ mean (±SD) age was 33.2 (±5.5) years. The majority of the mothers (53.6%) were university graduates, and approximately half (51%) were employed. Similarly, most fathers (58.2%) were university graduates, and nearly all (97.4%) were employed. About 20.3% of households consisted of five or more members. Additionally, 37.0% of the mothers had only one child, and 3.4% reported a history of child death.

On average, the mothers had 1.8 children with a standard deviation of 0.8. The children’s mean (±SD) age was 3.9 (±1.3) years, and 52.3% were male. The average (±SD) birth weight of the children was 3174.5 (±499.8) grams. Among the participants, 6.6% of the children had a low birth weight (<2500 g), and 7.5% were born prematurely. Additionally, half of the children (50%) attended nursery or kindergarten (Table 1).

Among the 878 participants, 15.8% of the mothers, 24.0% of the children, and 5.1% of mother–child pairs had at least one chronic illness. Conversely, the majority (65.3%) of mother–child pairs were both healthy (Table 1).

The breakdown of chronic illnesses among the children in our study is as follows: 31.6% (*n* = 67) with allergic diseases (including asthma), 15.1% (*n* = 32) with malignancies, 15.1% (*n* = 32) with neurological diseases, 7.5% (*n* = 16) with congenital conditions, 6.6% (*n* = 14) with hematological diseases, 5.7% (*n* = 12) with nephrological diseases, 4.7% (*n* = 10) with endocrinological diseases, 4.2% (*n* = 9) with rheumatological diseases, 2.8% (*n* = 6) with metabolic diseases, 2.8% (*n* = 6) with immunological diseases, 2.4% (*n* = 5) with gastroenterological diseases, and 1.4% (*n* = 3) with pulmonary diseases.

As for the chronic illnesses among the mothers, the distribution is as follows: 40% (*n* = 50) with endocrinological diseases, 12.8% (*n* = 16) with allergic diseases, 12% (*n* = 15) with neurological diseases, 11.2% (*n* = 14) with rheumatological diseases, 8.8% (*n* = 11) with cardiac diseases, 5.6% (*n* = 7) with hematological diseases, 4% (*n* = 5) with malignancies, 4% (*n* = 5) with gastroenterological diseases, and 1.6% (*n* = 2) with nephrological diseases.

### Parental Attitude Scale

The median values for the parental attitude subscale scores are seen in Table 1.

It was observed that a high democratic PAS score was not associated with the health status of the mother or the child. The percentage of mothers with high democratic PAS scores was higher in young mothers (<30 years), higher among mothers who had no job, and more prevalent in mothers with children aged 2–3 years (*p* = 0.034, *p* = 0.014, *p* = 0.014, respectively, Table 2).

No association was found between a high authoritarian score and the health status of the mother or child or other maternal and child characteristics (Table 2).

**Figure 1:**
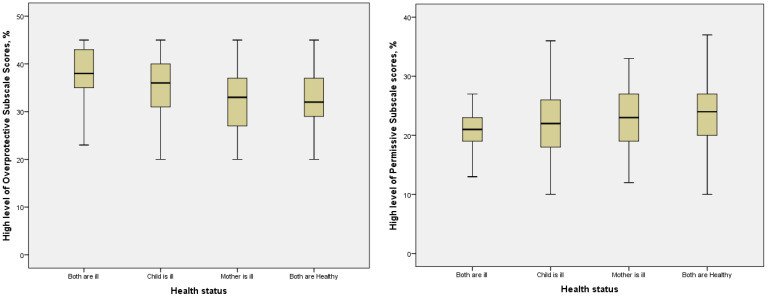
Boxplot (including minimum, first-quartile, median, third-quartile, and maximum values) of percentages of high overprotective and permissive subscale scores according to the health status of mother–child pairs.

The percentage of mothers with a high overprotective PAS score was statistically higher when both the mother and child were ill, as well as when only the child was ill, compared to situations where only the mother was ill or both were healthy (*p* < 0.001, Figure 1, Table 2). As maternal education and age increased, the percentage of mothers with high overprotective PAS scores decreased. Higher percentages of mothers with high overprotective PAS scores were observed among non-working mothers, those in extended families, those with more than one child, those who aged 2–3 years, those whose child is attending kindergarten home care, and those with a term birth, compared to their counterparts. Mothers with a history of infant or child loss had higher overprotective scores, while intrauterine loss had no significant impact (Table 2).

The percentage of mothers with high permissive PAS scores was higher when the mother was ill or when both were healthy, compared to cases where both were ill (Figure 1). Factors such as maternal age, education level, employment status, family structure, pregnancy duration, and birth weight influenced high permissive scores. Mothers with a history of intrauterine loss had higher permissive scores compared to other groups (Table 2).

Multiple logistic regression analysis revealed no significant relationship between the democratic and authoritarian scores and any of the mother–child variables.

After adjusting for maternal age, education, employment status, family structure, number of children, child’s age, pregnancy duration, birth weight, child care, and fetal/child loss in the family, having a sick child was associated with a 1.6-fold increase in the likelihood of a high overprotective PAS score, and both the mother and child being ill showed a 2.94-fold increase. The same relationship was confirmed when other PAS subscales were included in the model (Table 3).

Similarly, after adjusting for the same confounding factors, the likelihood of a high permissive score was 2.56 times lower (OR = 0.39, 95% CI: 0.15–1.01) when both mother and child were ill compared to when both were healthy. In Model 2, which included other PAS subscales as confounders, this association was confirmed with OR = 0.37 (95% CI: 0.14–0.99) (Table 3).

## 4. Discussion

The data collected in this study supported the hypothesis that mothers exhibit in-creased levels of overprotectiveness when the child is ill, regardless of whether the mother is also affected by illness. In contrast, the mother’s own chronic illness did not significantly influence overprotective behaviors when the child is healthy. Additionally, permissive scores were lower when both the mother and child were ill compared to situations where both were healthy. However, the findings indicated that authoritarian and democratic parenting styles remained stable, suggesting a nuanced understanding of maternal attitudes in these challenging circumstances. 

### 4.1. Overprotective Attitudes

In our study, a significant finding emerged regarding the relationship between maternal and child chronic illness and overprotective behaviors. When both the mother and child experienced chronic illnesses, and when only the child was ill, overprotective scores increased notably. Our findings emphasize how the dual presence of chronic illness can exacerbate maternal stress and influence protective tendencies. This aligns with the understanding that mothers often bear the primary responsibility for caring for ill children, which can lead to heightened anxiety and a tendency to exhibit overprotective behaviors [33]. Pinquart’s extensive meta-analysis, including 325 studies of 31,288 children with a chronic illness, revealed higher levels of overprotection [34]. Research indicates that the parents of chronically ill children typically display more controlling and directive behaviors compared to those with healthy children [17,35]. Heightened parental protectiveness may serve as an adaptive response to safeguard the health of their children while managing their own stress. However, this well-meaning protectiveness can transition into overprotection, making it difficult for parents to balance their child’s well-being and autonomy [3,36]. Parental overprotection can adversely affect a child’s adjustment, often resulting in behaviors that exceed the child’s developmental stage and coping abilities [36]. For instance, a study focused on hemophilia patients revealed that mothers often adopted overprotective attitudes out of fear of bleeding incidents, leading to restrictions that minimized their children’s opportunities for independent experiences [37]. Furthermore, parental overprotection can hinder the development of self-efficacy in children, resulting in feelings of overwhelm and reduced assertiveness [38]. Holmbeck et al. found a correlation between parental overprotection and diminished behavioral autonomy, alongside increased externalizing of problems in children with chronic illnesses [39]. In a study by Jakubowska-Winecka et al. study on parental attitudes and medication adherence in adolescents after liver and kidney transplantation, an overprotective attitude positively affected medication adherence and cooperation during treatment. It involved the highest medication adherence, regardless of disease type, but also a lower level of knowledge about the disease and a lower involvement of the patient in the treatment process. It is understandable that in the event of a serious chronic disease, the parents show a natural tendency to intensify their efforts to protect their child [40]. High parental protection and control can be beneficial when strict treatment adherence is crucial until the child’s self-care skills and own sense of health responsibility improve [18]. Balancing a child’s well-being and fostering independence are crucial in families facing chronic illness. When both mother and child have chronic conditions, increased stress may prompt overprotectiveness. Interventions addressing this can boost family resilience amidst chronic illness. On the contrary, Alkan et al. [41] reported no significant differences in overprotective attitudes between parents of children with congenital heart disease and those of healthy children.

### 4.2. Permissive Attitudes

Our findings regarding permissive parental attitudes revealed significant differences in both mother and child illness, comparing groups of both healthy and only mother’s illness. Permissive parenting is characterized by a lack of structure and rules, inconsistency in discipline, excessive gift-giving, and a tendency to avoid conflict while prioritizing friendship over authority [42]. However, in the context of chronic illness, this style may also lead to challenges in setting appropriate boundaries and expectations [43]. Research indicates that inconsistent disciplinary approaches as a result of permissive attitudes potentially complicate the management of health-related behaviors in children [44]. For parents of chronically ill children, the desire to accommodate and support their child may inadvertently lead to a lack of structure, which can hinder the development of necessary self-management skills [36]. In this regard, balancing a nurturing approach with clear guidelines becomes crucial, particularly as children navigate the complexities of living with chronic conditions [4]. The existing literature suggests that permissive parenting can have both positive and negative implications, especially in families managing chronic health issues [4,44,45]. A further exploration of how these dynamics interact could provide valuable insights into fostering healthier family environments and supporting child development in the context of chronic illness.

### 4.3. Democratic and Authoritarian Attitudes

In our study, we did not find a significant association between maternal–child health and democratic and authoritarian attitude scores. This aligns with the findings of Alkan et al. [41], who reported no significant differences in democratic or authoritarian attitudes between the parents of children with congenital heart disease and those of healthy children. In contrast, a study on mothers of children with epilepsy found significantly lower democratic attitude scores and higher authoritarian attitude scores compared to a control group [46]. The study by Avcil et al. [47] highlighted that mothers of pediatric asthma patients exhibited lower democratic attitude scores, which was attributed to a perceived lack of support and shared decision-making with their children. Qui et al. [33] found that parents of children with chronic illnesses often reported lower levels of authoritative (democratic) parenting. Pinquart’s extensive meta-analysis found higher levels of authoritarian and neglectful parenting in children with a chronic illness, as well as lower levels of democratic parenting compared with families with healthy children [34]. They concluded that most families with a child with chronic physical illness adapt well with regard to the parent–child relationship and parenting behaviors/styles. However, they reported that some families of children with specific diseases—such as epilepsy, hearing impairment, and asthma—may have difficulties finding appropriate levels of protective behaviors, control, and parental warmth and building positive relationships between parents and children [34]. This indicates that mothers of children with epilepsy may struggle to establish supportive relationships with their children, leaning towards increased authoritarianism and reduced democratic attitudes, likely due to safety concerns and anxiety about seizures.

### 4.4. Group Where Only the Mother Is Ill

We did not find a significant difference in democratic, authoritarian, overprotective, or permissive parental attitude scores in the group where only the mother was ill when compared with both being healthy. Previous studies have reported that children and adolescents of ill parents are vulnerable to a range of negative psychosocial outcomes, such as higher rates of depression, anxiety, and behavioral problems, alongside lower self-esteem and social competence [48,49,50]. However, none of these studies investigated the parental attitudes of chronically ill parents.

### 4.5. Mother–Child Characteristics and Parenting

Non-working mothers demonstrated higher overprotective scores compared to their working counterparts. These findings may stem from lower self-confidence among less educated and unemployed mothers, particularly in anticipating and managing the challenges associated with chronic diseases. Lower education levels and unemployment are often linked to restricted access to information and resources, which can heighten feelings of insecurity when addressing a child’s chronic illness. It is crucial for healthcare professionals to acknowledge these factors, as addressing limited confidence and information access can empower mothers, fostering healthier parent–child relationships in the face of chronic illness challenges.

Our analysis also indicated that mothers with only one child, those whose children were aged 4 or older, or those who were employed tended to exhibit more permissive attitudes. This trend may reflect the growing independence of older children, who often take on more responsibility for managing their illnesses, thereby reducing the need for parental control and protection [35]. Our study highlights the evolving nature of parental attitudes, which adapt to meet the shifting needs and abilities of children at different ages. By recognizing the impact of child age and family dynamics on parental attitudes, healthcare professionals can tailor support and interventions to better address the needs of families grappling with chronic illness. This comprehensive approach fosters a nurturing environment conducive to optimal development and well-being in both children and parents.

Additionally, we observed a significant decrease in the mean scores for overprotective, attitude among mothers with children attending nursery or kindergarten (*p* < 0.001). Mothers may intentionally reduce overprotective tendencies to help their children adjust to the school environment and foster independence. Encouraging children to manage daily tasks independently can enhance self-confidence and resilience, preparing them for the challenges that they may face at school. Overall, attendance at nursery or kindergarten appears to influence maternal parenting attitudes, promoting a balanced and adaptive approach that aids in child development and adaptation to new settings.

In our study, although univariate analyses showed that mothers who experienced child loss were more overprotective compared to those who did not experience loss or had an intrauterine loss, this was not observed in the multiple logistic regression. On the other hand, mothers of individuals who experienced sibling loss during the perinatal/postnatal period were reported to have higher protective scores [51]. In our study, those who experienced intrauterine loss had higher permissive scores compared to the other groups. As reported previously, parents who were initially overprotective may have become more permissive after the loss, as such a significant event can alter one’s values, which in turn affects parenting behaviors [52]. A qualitative study on the families of victims of the Sewol ferry disaster in South Korea showed that parents exhibited neglectful or permissive parenting behaviors, which promoted independence [53].

### 4.6. Strengths and Limitations

This study provides a comprehensive overview of the distribution of chronic illnesses among children and mothers, encompassing a wide range of medical conditions across various organ systems. By incorporating diverse chronic illnesses, it offers valuable insights into the health challenges faced by both groups, facilitating a nuanced understanding of their healthcare needs. The stratification of participants into four distinct groups enables a detailed exploration of how chronic illness impacts family dynamics and parental caregiving, thereby informing intervention strategies for families confronting the challenges associated with childhood chronic illness. Furthermore, the substantial sample size, which includes a significant number of children and mothers with chronic illnesses, strengthens the data for analysis and interpretation. The involvement of three centers with varying socioeconomic conditions enhances the study’s applicability and relevance across different contexts.

However, this study has several limitations. One notable limitation is the reliance on self-reported data in identifying chronic illnesses in both children and mothers, which may introduce bias due to under-reporting or over-reporting. Additionally, the data collection was limited to the mother–child dyad, omitting the evaluation of father–child or mother–father communication. Mothers were primarily chosen for the assessment of parental attitudes because they often allocate more time to their children and are more responsible for medication and treatment decisions. Moreover, mothers may experience a greater impact from the child’s illness, as they frequently adjust their employment status to provide full-time care, while fathers typically continue working outside the home [54]. To gain a more holistic understanding of family dynamics, future studies should examine the relationship between chronic illness and parental attitudes in both fathers and mothers.

Another limitation is that more than half of the participants in the study were college-educated, which may limit the generalizability of the findings across the full spectrum of parental education and socioeconomic status.

Additionally, the focus on chronic illnesses at a specific point in time restricts our understanding of factors affecting disease progression, treatment adherence, and health outcomes. Longitudinal data on illness progression would provide valuable insights into disease management and deepen our understanding of the interplay between parenting styles and chronic illness. Overall, while this study contributes significantly to the literature, further research is needed to address these limitations and enhance our understanding of the complex dynamics involved in parenting a child with a chronic illness.

## 5. Conclusions

This study reveals that when a child is affected by a chronic illness—whether or not their mother is also ill—mothers tend to exhibit higher levels of overprotection and lower levels of permissiveness, while their levels of authoritarianism and democratic attitudes in parenting remain relatively stable. Our findings suggest that interventions aimed at strengthening the parent–child relationship should prioritize the addressing of overprotective behaviors over responsiveness or demandingness. Effective interventions can assist parents in finding a more balanced approach that meets their child’s autonomy needs while effectively managing the illness. Furthermore, these results underscore the importance of physicians caring for children with chronic diseases assessing both the presence of the illness and the parenting attitudes of mothers, particularly in relation to treatment compliance. By recognizing and leveraging family strengths, primary care physicians can enhance treatment outcomes and improve overall family functioning through tailored interventions and support services.

## Figures and Tables

**Table 1:** General characteristics of mother–child pairs.

Family Characteristics	Mean ± SD, Median (25–75 p), [Min–Max], N (%)	Characteristics	Mean ± SD, Median (25–75 p), [Min–Max], N (%)
**Mother’s age,** years	33.2 ± 5.5 [19–52]	**Number of children**	2 [1–7]
<30 years	232 (26.4)	1 child	325 (37.0)
30–34 years	301 (34.3)	>1 children	553 (63.0)
35–39 years	223 (25.4)	**Gestational duration** < 37 w	66 (7.5)
≥40 years	122 (13.9)	**Birth weight,** grams	3175 ± 500 [610–5000]
**Mother’s education**		<2500	58 (6.6)
≤Secondary school	166 (18.9)	≥2500	820 (93.4)
High school	241 (27.4)	**Child’s sex,** male	459 (52.3)
University	471 (53.6)	**Child’s age,** year	3.9 ± 1.3 [2–6]
**Mother has a job**	448 (51.0)	2	156 (17.8)
**Household size**	4.0 ± 1.0 [3–11]	3	211 (24.0)
<5 person	700 (79.7)	4	208 (23.7)
≥5 person	178 (20.3)	5	187 (21.3)
**Family type,** nuclear	787 (89.6)	6	116 (13.2)
**History of fetus or child death**	30 (3.4)	**Nursery/kindergarten care**	
Intrauterine loss	14 (1.6)	Yes	439 (50)
Infant or child death	16 (1.8)	No	439 (50)
**Mother–child health status**		**Parental Attitude Scale**	
Only mother is ill	94 (10.7)	Democratic score	74 (69–79)
Only child is ill	166 (18.9)	Authoritarian score	19 (17–23)
Both are ill	45 (5.1)	Overprotective score	33 (29–38)
Both are healthy	573 (65.3)	Permissive score	23 (20–27)

**Table 2:** Relationship between mother–child characteristics and high scores in parental attitudes.

	Overall	Democratic Score ≥ 78		Authoritarian Score ≥ 23		Overprotective Score ≥ 37		Permissive Score ≥ 27	
	*n* (%) *	% **	*p*	% **	*p*	% **	*p*	% **	*p*
**Overall**	878 (100)	33.4		25.4		33.9		27.4	
**Health Status**			0.245		0.893		<0.001		0.018
Both are ill	45 (5.1)	40.0		24.4		62.2 ^a^		13.3 ^a^	
Only child is ill	166 (18.9)	38.0		26.5		47.0 ^a^		21.1 ^ab^	
Only mother is ill	94 (10.7)	36.2		22.3		28.7 ^b^		29.8 ^b^	
Both are healthy	573 (65.3)	31.1		25.7		28.8 ^b^		30.0 ^b^	
**Mother’s age**			0.034		0.937		0.002		0.036
<30 years	232 (26.4)	40.9 ^a^		25.4		44.0 ^a^		25.0 ^a^	
30–34 years	301 (34.3)	31.6 ^b^		24.6		31.9 ^b^		23.9 ^a^	
35–39 years	223 (25.4)	30.9 ^b^		26.9		29.6 ^b^		29.6 ^ab^	
≥40 years	122 (13.9)	27.9 ^b^		24.6		27.9 ^b^		36.9 ^b^	
**Mother’s education level**			0.544		0.190		<0.001		<0.001
≤ Secondary school	166 (18.9)	31.3		30.7		63.9 ^a^		18.7 ^a^	
High school	241 (27.4)	36.1		25.3		41.5 ^b^		16.6 ^a^	
University	471 (53.6)	32.7		23.6		19.5 ^c^		36.1 ^b^	
**Mother has a job**			0.014		0.872		<0.001		<0.001
Yes	429 (48.9)	29.4		25.6		19.1		39.2	
No	449 (51.1)	37.2		25.2		48.1		16.3	
**Family type**			0.429		0.977		<0.001		0.006
Nuclear	787 (89.6)	33.8		25.4		31.8		28.8	
Extended	91 (10.4)	29.7		25.3		52.7		15.4	
**Number of children**			0.157		0.125		0.024		0.091
1 child	325 (37.0)	36.3		22.5		29.2		30.8	
>1 children	553 (63.0)	31.6		27.1		36.7		25.5	
**Child’s age**			0.014		0.670		0.013		0.159
2–3 years	365 (41.6)	38.1		24.7		38.6		24.9	
4–6 years	513 (58.4)	30.0		25.9		30.6		29.2	
**Kindergarten attendance**			0.830		0.103		<0.001		0.496
No	439 (50)	33.0		27.8		43.3		26.4	
Yes	439 (50)	33.7		23.0		24.6		28.5	
**Gestational duration**			0.995		0.944		0.020		0.041
<37 weeks	66 (7.5)	33.4		25.4		32.9		28.3	
≥37 weeks	812 (92.5)	33.3		25.8		47.0		16.7	
**Birth weight**			0.294		0.479		0.017		0.016
<2500 g	58 (6.6)	39.7		29.3		48.3		13.8	
≥2500 g	820 (93.4)	32.9		25.1		32.9		28.4	
**History of fetus or child death**			0.451		0.942		0.012		0.042
No	848 (96.6)	33.6		25.5		33.3 ^a^		27.0 ^a^	
Intrauterine loss	14 (1.6)	35.7		21.4		35.7 ^ab^		57.1 ^ab^	
Infant or child death	16 (1.8)	18.8		25.0		68.8 ^b^		25.0 ^a^	

^a,b,c^ Values with different letters in the same column are significantly different from others (*p* < 0.05); * column percentage; ** row percentage.

**Table 3:** Association between mother–child characteristics and high scores in overprotective and permissive parental attitudes: a multiple logistic regression analysis.

	Overprotective Score ≥ 37 Model 1		Overprotective Score ≥ 37 Model 2		Permissive Score ≥ 27 Model 1		Permissive Score ≥ 27 Model 2	
	AOR	95% CI	AOR	95% CI	AOR	95% CI	AOR	95% CI
**Health status**								
“Only mother is ill” vs. “Both healthy”	0.82	0.48–1.38	0.78	0.46–1.33	1.26	0.76–2.11	1.31	0.79–2.20
“Only child is ill” vs. “Both healthy”	1.60	1.08–2.39	1.56	1.04–2.34	0.83	0.53–1.31	0.84	0.53–1.31
“Both are ill” vs. “Both healthy”	2.94	1.45–5.97	2.90	1.42–5.89	0.39	0.15–1.01	0.37	0.14–0.99
**Mother’s age**								
30–34 vs. <30 years	0.92	0.61–1.37	1.05	0.69–1.60	0.60	0.39–0.94	0.60	0.38–0.93
35–39 vs. <30 years	1.10	0.69–1.75	1.24	0.77–2.01	0.67	0.41–1.09	0.65	0.40–1.06
≥40 vs. <30 years	0.84	0.48–1.47	0.95	0.53–1.69	1.03	0.60–1.76	1.00	0.59–1.72
**Mother’s education level**								
High school vs. ≤ Secondary school	0.52	0.34–0.80	0.50	0.32–0.77	0.67	0.38–1.16	0.73	0.41–1.27
University vs. ≤ Secondary school	0.29	0.17–0.47	0.25	0.15–0.42	1.02	0.57–1.81	1.17	0.65–2.11
**Mother has a job**								
Yes vs. no	0.54	0.36–0.81	0.53	0.35–0.80	2.93	1.90–4.53	2.99	1.92–4.66
**Family type**								
Extended vs. Nuclear	1.75	1.07–2.87	1.93	1.17–3.19	0.47	0.25–0.88	0.45	0.24–0.84
**Number of children**								
>1 children vs. 1 child	1.10	0.77–1.55	1.11	0.78–1.58	0.83	0.59–1.17	0.81	0.58–1.15
**Child’s age**								
4–6 vs. 2–3 years	1.03	0.71–1.49	1.07	0.74–1.56	1.06	0.76–1.48	1.05	0.75–1.47
**Gestational duration**								
<37 weeks vs. ≥37 weeks	0.95	0.44–2.02	1.05	0.49–2.28	1.03	0.40–2.68	1.00	0.38–2.60
**Birth weight**								
<2500 vs. ≥2500 g	1.54	0.69–3.45	1.46	0.64–3.33	0.36	0.12–1.06	0.37	0.12–1.11
**History of fetus or child death**								
Intrauterine loss vs. no	1.27	0.38–4.24	1.08	0.30–3.89	4.33	1.37–13.72	4.28	1.34–13.68
Infant or child death vs. no	1.56	0.49–5.03	1.87	0.56–6.24	2.05	0.59–7.09	1.78	0.50–6.31
**Kindergarten attendance**								
Yes vs. no	0.65	0.45–0.94	0.62	0.42–0.90				
**Democratic subscale**								
Score ≥78 vs. <78			2.53	1.81–3.55			0.71	0.50–1.02
**Authoritarian subscale**								
Score ≥23 vs. <23			1.26	0.87–1.81			1.12	0.78–1.61
**Overprotective subscale**								
Score ≥37 vs. <37							1.44	0.98–2.12
**Permissive subscale**								
Score ≥27 vs. <27			1.38	0.94–2.03				
Constant	1.39		0.83		0.36		0.32	

## Data Availability

The data presented in this study are available on request from the corresponding author due to ethical considerations.
